# Toward a typology of health-related informal credit: an exploration of borrowing practices for paying for health care by the poor in Cambodia

**DOI:** 10.1186/1472-6963-12-383

**Published:** 2012-11-07

**Authors:** Por Ir, Bart Jacobs, Bruno Meessen, Wim Van Damme

**Affiliations:** 1 National Institute of Public Health, Ministry of Health, PO BOX 1300, Phnom Penh, Cambodia; 2 Department of Public Health, Institute of Tropical Medicine, Antwerp, Nationalestraat, Antwerp, 155, B-2000, Belgium; 3 Health Sector Support Programme, Luxembourg Development, Ministry of Health, PO BOX 7084, Vientiane, Lao PDR

**Keywords:** Borrowing practices, Indebtedness, Typology, Informal credits, Cambodia

## Abstract

**Background:**

Borrowing money is a common strategy to cope with health care costs. The impact of borrowing on households can be severe, leading to indebtedness and further impoverishment. However, the available literature on borrowing practices for health is limited. We explore borrowing practices for paying for health care by the poor in Cambodia and provide a typology, associated conditions, and the extent of the phenomenon.

**Methods:**

In addition to a semi-structured literature review, in-depth interviews were conducted with representatives of 47 households with health-related debt and 19 managers of formal or informal credit schemes.

**Results:**

A large proportion of Cambodians, especially the poor, resort to borrowing to meet the cost of health care. Because of limited cash flow and access to formal creditors, the majority take out loans with high interest rates from informal money lenders. The most common type of informal credit is locally known as *Changkar* and consists of five kinds of loans: short-term loans, medium-term loans, seasonal loans, loans for an unspecified period, and loans with repayment in labour, each with different lending and repayment conditions and interest rates.

**Conclusion:**

This study suggests the importance of informal credit for coping with the cost of treatment and its potentially negative impact on the livelihood of Cambodian people. We provide directions for further studies on financial protection interventions to mitigate harmful borrowing practices to pay for health care in Cambodia.

## Background

### Introduction

Borrowing is a common strategy to cope with the costs of illness. Kruk and colleagues [1] analysed the results of the World Health Survey for forty low- and middle-income countries that together represent more than half the global population and found that 22% of households had borrowed money to cope with the cost of illness. Such coping mechanisms are most likely to occur in countries where social health protection schemes are underdeveloped or do not encompass informal sector workers [2]. Russell and Gilson [3] reported from Sri Lanka that health expenditure equalling 2.5% to 5% of monthly income triggered borrowing. Coping with health care payments through borrowing and selling assets is found to be common in many African countries, ranging from 23% of households in Zambia to 68% in Burkina Faso [4].

The 2010 Cambodian Demographic and Health Survey found that 18% of people who sought care for an illness resorted to borrowing. For serious illnesses, the proportion of borrowing was as high as 36% [5]. Murshid [6] studied 244 households located in three villages of diverse agro-ecological areas in Cambodia and found that 20% of the poorest socioeconomic quintile borrowed money to pay for health related costs versus 9% of the best-off with average annual interest rates of 170% and 53% respectively. In a recent survey among 5,395 households, 58% were found to be in debt. Of the indebted, 30% had more than one loan. Worryingly, 33% of loans were taken out for paying medical bills [7]. Further analysis of the same data set indicated that of the poorest tercile households that experienced a health shock, 26% resorted to borrowing money with interest to pay for the treatment, compared with 9% of the best-off tercile households [8].

Health equity funds (HEF) –third party mechanisms reimbursing selected health care providers for curative services rendered to eligible poor [9,10] –were developed in Cambodia to facilitate access to health care for the poor. Although such funds reduced the incidence of borrowing for health care in an urban slum in Phnom Penh [11], many poor households eligible for such scheme still had to borrow. To assess the effects of user fee exemptions through HEF on health seeking behaviour, out-of-pocket expenditure and coping mechanisms, hospitalised exempted and paying patients were matched and interviewed in two separate studies at one rural hospital. In the first study [12], of the 199 exempted patients, 83% still borrowed money. They borrowed an amount on average 3.4 times the sum of direct costs incurred during the illness episode. Respective figures for paying patients were 48% and 0.74. In the second study [13], 82% of exempted patients resorted to borrowing; on average they borrowed 6.6 times the total amount of direct costs. Conversely, only 35% of paying patients borrowed and the amount borrowed was 56% of the sum of direct costs associated with the concerned illness. The excessive amounts borrowed (in relation to direct costs) by exempted patients are thought to be due to opportunity costs incurred when hospitalised.

Loans taken out for medical care differ from investment loans as they often respond to economic stress events that require lump sums which need to be paid urgently, with relatively little time for informed decision-making concerning available credit suppliers. This is especially the case when access to formal credit suppliers such as banks is limited because of many constraints, including scarce collateral, market segmentation and lack of effective communication [14]. In this context, households in need of urgent cash often borrow from unregulated money lenders who charge usurious interest rates [15]. While borrowing can help households to faster access health care in case of illness [16], taking out loans in such situation often leads to the sale of productive assets and results in impoverishment. For example, in East Asia, the inability to repay loans can lead to debt bondage, literally enslaving people [17]. Van Damme *et al*. [18] followed up people who had borrowed to pay costs associated with treatment of dengue in Cambodia and found that 62% of them were still paying off their debt and its interests after one year. Other studies in Cambodia showed that costs related to illness are a major cause of sale of land to repay illness-related debts. Moreover, when failing to repay such debts, confiscation of assets is often the result [15,19].

A better understanding of the process through which Cambodian households obtain cash for paying for health care and associated lending and repayment conditions will help to define appropriate policies and interventions to limit the negative impact of such practices. Surprisingly little has been published in the health literature on this issue so far. We contribute to filling this knowledge gap by exploring practices of borrowing for paying for health care by the Cambodian population, predominantly the poor, in an attempt to provide examples of available health-related informal credit and associated conditions.

### Socio-economic and health context in Cambodia

Cambodia is a low income country with a GDP of US$795 in 2010 [20] and a population of 13.4 million people (2008 census). Over 80% of the population are rural-based and 30% live below the absolute poverty line of US$0.59 per day [21]. The country is recovering from three decades of civil strife, including Maoist-style social experiments by the Khmer Rouge regime under which up to a quarter of the population were killed or died of starvation. As a result, the existence of a State with a more or less functioning public administration is fairly recent –not much more than 20 years –while there remains a considerable shortage of people with the required technical and professional skills [22]. Comprehensive public health reforms started in 1996 when the ministry of health launched its Health Coverage Plan and impressive gains in health outcomes have been attained since [5]. Nevertheless, private-for-profit health providers, many of them informal, dominate the health care market and are the preferred option for the population [23].

### Credit suppliers in Cambodia

The formal credit suppliers in Cambodia include banks and microfinance institutions (MFIs) that are recognised by the National Bank of Cambodia. According to the “Law on Banking and Financial Institutions” enacted in November 1999, the government recognizes three categories of banking institutions: (1) commercial banks, which require a minimum registered capital of US$13 million and can carry out all banking activities; (2) specialized banks, which require a minimum registered capital of US$2.5 million and can carry out a limited number of banking activities, as specified in the terms of their license; and (3) MFIs, which require a minimum registered capital of approximately US$62,500. The latter provide financial services to entrepreneurs and small businesses that have limited access to banking services. Organisations that provide credit to the rural population but do not meet the above criteria can only register as rural credit operators.

Banks are mainly located in towns, contrary to MFIs and rural credit operators. By 2011 there were 20 licensed MFIs and over 20 rural credit operators in Cambodia that had US$492 million in outstanding loans for about one million clients [24]. For individual loans collateral is required, but for group loans this is not the case. However, because of social exclusion, the poor and vulnerable are often unable to participate in group activities and tend to lack collateral; hence, they are not necessarily reached by these formal creditors [25].

Along with these formal credit suppliers, there are widespread informal or non-institutional credit suppliers such as individual money lenders, groups of mutually organized individuals and partnership. Individual money lenders include relatives, friends, neighbours, landlords, market vendors, traders and ‘informal’ money lenders. The latter are ‘professional’ individual money lenders, but unregulated, non-subsidized and often charge usurious interest rates, though easily accessible with little or no collateral requirements. In addition, there are semi-formal credit suppliers, which are local development organizations, including rice banks and pig banks, making them unsuitable for emergency loans.

## Methods

### Data collection

Because of the paucity of published documents in the health literature on borrowing practices for paying for health care, literature from other sectors, including rural development and poverty alleviation, was consulted in a thorough but non-systematic manner. For this purpose Google Scholar was consulted in addition to PubMed using the words ‘credit’, ‘formal’, informal’, ‘rural’, ‘debt payment’, ‘interest rates’, ‘loans’, ‘borrowing practices’ and ‘rural finance’, alone or in combination. A similar search was done on the ELDIS website, while for Cambodia documents on rural development by the Cambodia Development Resource Institute were consulted. From the references further documents were traced if the title appeared relevant.

To better understand existing sources of credit and their associated practices as well as their impact on livelihoods in Cambodia, in-depth interviews using a semi-structured questionnaire were conducted with 47 representatives of households who reported health-related debts. These interviews were done in the local vernacular and took place at the residence of the respondents or at the hospital between August and October 2006.

Households with health related debt were purposively selected among the residents in Thmar Pouk and Sotnikum rural districts and urban slums in Siem Reap town and Phnom Penh capital. In each study site, there was a HEF scheme. The operators of HEF had good knowledge about poor households in their catchment area and about those who experienced health-related debt. Through these operators initial interviewees were identified. The latter were then asked to identify other indebted households with similar socio-economic status. Questions concerned household characteristics, degree of indebtedness and associated amounts, the respective health problem for which loans were sought, care seeking behaviour, borrowing practices, source(s) of credit and related conditions, and coping mechanisms.

Semi-structured interviews were also conducted with twelve managers of HEF schemes, microfinance institutions or banks, together with seven informal money lenders. The latter were mainly identified through the interviewed representatives of indebted households. All interviewees were based in the study sites. The interviews were done by three experienced researchers.

### Analytical framework and data analysis

**Figure 1:**
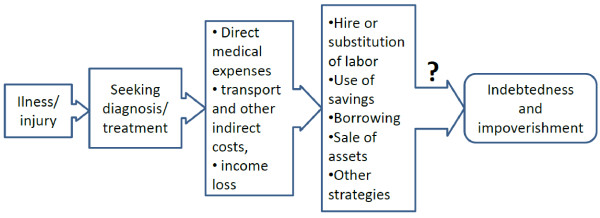
Simplified flow chart of key issues relating to the economic consequences of illness.

*Source: adapted from McIntyre et al.*[2]*.*

Development economics is sometimes described as economics applied to settings characterised by failure in different markets, including insurance and credit markets. For countries in economic transition like Cambodia, where markets are insufficiently regulated, market liberalisation can create economic risks for households, especially in the health sector [26,27]. As pointed out by Meessen *et al*. [26], economic transition can lead to economic growth, but also reshape the pattern of entitlements (e.g. from universal access to free health care to access according to ability to pay), and change demand for and supply of health care. The epidemiological and demographic transitions together with asymmetry of information and limited regulatory constraints often lead to increased demand for and supply of unnecessary and expensive care. When coupled with insufficiently developed social health protection schemes, severe economic consequences for households can arise when obtaining health care. This occurs through a complex and interactive process which can be simplified as in Figure 1.

As presented earlier in this paper, borrowing money is a common strategy to meet the cost of health care in many low- and middle-income countries. Because formal creditors are unlikely to be used for emergency loans to meet the costs of illness, we focus on informal creditors. Phlong [25] differentiates four types of informal credit in Cambodia: (1) *Khchey* –loans between friends and neighbours without interest rates; (2) *Tontine* – an informal rotating group saving and lending system with 5 to 50 participants; (3) *Bancham* –pawning which implies depositing a valuable item as collateral with the moneylender; and (4) *Changkar* –money lending with interest by mainly informal money lenders and better-off individuals. It is this last type of informal lending which is of special interest and will be described in more detail, especially the types of loans that they extend and related payment conditions.

We assessed key characteristics of each type of *Changkar* (including lending source, amount of loan, lending period, lending conditions, interest rate, purposes of loan, and mode of repayment), their advantages and disadvantages, and their frequency of use for health care. Based on this information, mainly on the interest rate and repayment conditions, the level of associated economic risk or harm for the borrowers was estimated.

Interviews were tape recorded and transcribed. Resulting data were manually coded and grouped by themes according to the abovementioned key characteristics of *Changkar* for analysis. Information from interviews with representatives of indebted households was carefully interpreted and triangulated with responses of managers and informal money lenders.

To allow comparison between types of *Changkar*, monthly interest rates were estimated by dividing the total amount of interest by the amount borrowed and number of months for repaying, as reported by interviewed borrowers. This was triangulated with interest rates reported by interviewed lenders. All figures provided by the interviewees in Cambodian Riel or Thai Baht were converted to US$.

### Ethical consideration

This study is part of the “AIDS and Indebtedness” study, which received ethical approval from the National Ethic Committee for Health Research in Cambodia in February 2006 with reference number 016 NECHR and Ethical Committee of Antwerp University.

## Results

### Key characteristics of indebted households and their indebtedness

A total of 47 representatives of households indebted due to health care costs were interviewed. They were questioned about household characteristics, degree of indebtedness and associated amounts, the concerned health problem for which loans were sought, care seeking behaviour, borrowing practices, source(s) of credit and related conditions, and coping mechanisms.

Table 1 summarises key characteristics of the 47 indebted households and their indebtedness according to place of residence. About half of them were from rural areas, the rest were urban residents. The main sources of income were daily labour and small business for the urban group compared to farming and foraging for the rural group. Based on socio-economic assessment criteria of HEF, they were all poor households at the time of interview.

**Table 1:** Key characteristics of the indebted households and their indebtedness

**Key variables**	**Urban n = 24**	**Rural n = 23**	**All n = 47**
Male-headed households (%)	16 (67)	16 (70)	32 (68)
Mean number of household members (SD)	4.5 (1.8)	6.1 (2.2)	5.3 (2.2)
Mean number of dependents (SD)	2.8 (1.5)	4.2 (2.1)	3.5 (1.9)
*Households by main source of income (%)*			
Farming	0	20 (87)	20 (43)
Casual labourer	11 (46)	1 (4)	12 (26)
Small business	9 (38)	0	9 (19)
Other sources	4 (17)	0	4 (8)
No income	0	2 (9)	2 (4)
*Households by socio-economic status (%)*			
Very poor	10 (42)	7 (30)	17 (36)
Poor	13 (54)	15 (65)	28 (60)
Near-poor	1 (4)	1 (4)	2 (4)
Median (mean) of total debt in US$	138 (516)	70 (211)	125 (367)
Median (mean) number of outstanding loans	2 (2.7)	1 (1.8)	2 (2.3)
Median (mean) rate of monthly interests in %	10 (11)	7.0 (23.4)	10 (17)
*Main sources of debt (%)*			
Money lenders	15 (63)	10 (44)	25 (53)
Relatives/friends/neighbours	4 (17)	9 (39)	13 (28)
Microfinance organisations	3 (13)	1 (4)	4 (9)
Banks	1 (4)	2 (9)	3 (6)
Others	1 (4)	1 (4)	2 (4)

***SD*** = standard deviation.

The average amount of pending debt was largest in the urban group. The main sources of reported loans in both groups were money lenders (53%) and relatives/friends/neighbours (28%). Urban interviewees reported the highest monthly interest rate.

### Common types of informal credit and associated conditions

As mentioned by Phlong [25], loans with interest from informal money lenders or Changkar are mostly used for urgent and unanticipated financial requirements as they are relatively flexible and easily accessible with no or limited paper work. Often no collateral is requested except for big loans or for persons without permanent residence. A clear deadline for repayment is set though not strictly respected, as failure to respect the deadline does not necessarily result in severe sanctions but translates into harassment by the lender against the borrower to identify means to repay the debt, including sale of assets.

The *Changkar* include five different kinds of loans, each with proper requirements and implications (Table 2), as elaborated with examples below.

**Table 2:** Overview of informal credit types and associated conditions

**Type of credit and characteristics**	**Advantages and disadvantages**	Frequency of use for health care*	Level of economic risk**
Short-term loan (*Luy Roab*)		+++++	++++
· Short lending period: 10–20 days	· Quick, easy access; addressing urgent requirements		
· Relatively small amounts	· High interest rates coupled to daily repayment		
· Daily repayment			
· High interest (20%), to be paid as extra days of repayment			
· Common in areas with commercial activities			
· Usually no requirement for collateral or paper work			
Medium-term loan (*Luy Ruos*)		+++	+++
· Lending period few months to a year	· Large amount for relatively long period		
· Relatively big amount	· High interest rates and requirement for collateral, making it less accessible for poor people		
· Interest rates of 5-10% per month			
· Deadline for repayment			
· Collateral required as well as official transaction			
Seasonal loan		++	++
· In-between season lending: 6–10 months	· Easy access for farmers		
· Relatively big amounts	· Repayment follows the farming cycle which is convenient for rural people		
· Interest rates of 50-100% for the season	· Repayment in kind, just following harvest when prices are low		
· Repayment after harvest, in crops			
· Very common Simple or no conditions				
Loans for unspecified period (*Luy Ngoab or Luy Chho*)		++++	++	
· Unspecified period	· Easy access for the poor			
· Variable amount	· Absence of repayment deadline is favourable for poor people			
· Relatively high interest: 1%/day; 5-30%/month	· High interest rates,			
	· requirement for collateral for big amounts			
· Flexible timing for repayment				
· Collateral for large amounts				
Loans with repayment in labour (*Yok Dai*)		+	+	
· Variable, though mostly short, lending period	· Easy access, especially for the poorest			
· Relatively small amount	· No money required, independent of cash flow			
· Interest of 100% for whole period	· Undervalued labour			
· Repayment in labour valued at 50%	· Hampers foraging on which poor depend for survival			
· Only available in rural areas	· High interest rates			
· No lending conditions				

* rated from the less frequent ‘+’ to the most frequent ‘+++++’; ** rated from the lowest ‘+’ to the highest ‘++++’.

**Short-term loans** cover a very short time period, typically between 10 and 20 days, though exceptionally 60 days or more. The amount is relatively small, often less than USD100. The payment is done through daily instalments of a fixed amount till the total amount is paid off with a few extra days for the interest (this may not be explicitly seen by some borrowers as interest). The interest often amounts to 20% of the loan. These loans are called ‘*Luy Roab*’ (counting money) in the vernacular and are common in areas with business activity such as markets. Because of its accessibility, this type of borrowing is commonly used by the poor to meet their health care costs.

"An HIV-infected widow with a 4-year old child residing in a village in Siem Reap. She has no property and lives with her brother. Her husband died of AIDS 10 months before the interview. When he was still alive, he was shopping around to treat his disease, mainly consulting private health practitioners and traditional healers. He spent about US$5,000 for various treatments whereby they borrowed many times from different lenders. The last loan was US$200 obtained from a money lender in their village. The amount to pay back was US$240 with a daily repayment of US$20 during 12 days, including the 20% interest rate. Unfortunately, her husband died after which she sold her plot of land and finally her house to reimburse the debts. She still had a debt of US$500 when interviewed, 10 months past the deadline. The lenders came to ask her every day. She sells wine, which allows her to earn a maximum of US$0.5 a day. She receives free antiretroviral treatment from an NGO, enabling her to restore her health but is of the opinion that she will never be able to pay back her outstanding debts."

**Medium-term loans** are called ‘*Luy Ruos’* (living money). The period of lending ranges from several months to one year. The amount of this type of loans can be significantly bigger than the short-term loans, up to USD500 or more. Interest is to be paid monthly and the loan capital should be repaid as a lump sum by the agreed deadline. The monthly interest rate is usually between 5% and 10%. Because the amount of the loan is considerable, collateral is often required and an official transaction document such as a signed paper is made. However, with trustful relationships built from previous loans with the lender, some households can borrow without collateral, but often have to pay double amounts of interest instead. This kind of loan is not common for covering costs of illness because the procedures to obtain it are unfit for meeting urgent financial needs.

"A young couple living with 3 relatives in a slum in Phnom Penh. The wife used to work in a garment factory at US$40 per month. Because of a worsening chronic heart condition she stopped working. Her husband and brothers are daily labourers and can jointly earn about $5 per day. Her husband borrowed US$600 from a private money lender in the village to buy a motorbike and to pay for health related expenses of his wife (about 40% of the loan). It was a loan for 4 months at a monthly interest rate of 6%. Unfortunately, his motorbike was stolen; he put a pair of golden ear rings as collateral, estimated at US$40. The creditor confiscated them because he was unable to pay the monthly interest for almost two months at the time of interview."

**Seasonal loans** (no particular local name) are common among farmers who face cash constraints during certain periods, especially before planting season. The lending period ranges from six to ten months, typically till the next harvest. The interest rate for the whole lending period varies from 50% to 100%. The interest and the loan capital are repaid at harvest time in the form of rice, but calculated in monetary value. This mode of repayment is unfavourable to the borrower as it occurs just after the harvest, when the commodity price is low. Additionally, the borrower often does not realise the loan comes with such a high interest rate. Although this loan is commonly used for agricultural investment, few use it to pay for health care-related costs.

"A rural family of 9 people (parents with 7 children) with an income based on farming and foraging. The mother was admitted to the provincial hospital in Siem Reap for post-operative complications three months prior to interview. Her situation had not improved since. About US$500 was spent on hospital fees, transport, food and other immediate needs during the hospitalization. The money was borrowed despite benefiting from free hospitalisation through the health equity fund. To meet the above mentioned expenses they borrowed US$250 from a relative and another US$250 from a money lender, without putting collateral. Both loans are to be paid back at the following harvest. The relative did not charge interest while the money lender demands 50% for the whole period (Repayment of US$375 for the loan of US$250)."

**Loans for an unspecified period,** called *Luy Ngoab or Luy Chho* (death money or stand-by money), are provided until the borrower can repay. Interest has to be paid regularly on a daily, weekly or monthly basis and the interest rates depend on the lender and amount of loan with bigger loans tending to have lower interest rates. The daily interest rate is often 1%, while monthly interest rates vary from 5% to 30%. The amount of loan also varies from around USD25 for a loan with daily interest payment to USD400 for loans with weekly and monthly interest payments. This type of loan is commonly used by poor households to pay for health care, but often the sum of interest exceeds the amount borrowed.

"A rural middle class family of 6 people with three children employed and one at school. The children earn US$1-1.5 per day while the father gets US$1.25 -1.75 per day. The family had a plot of land valued at US$5,000, five hectares of rice land and 4 adult cows. The wife was diagnosed with diabetes one year before the interview. Initially she was hospitalised at the district hospital after which she sought care from private providers in town. They sold 4 cows for US$500 and twice rice land for a total of US$475 and borrowed US$250 from a money lender to pay for her health care and other related costs. The loan was without collateral or deadline but came with a monthly interest rate of 5%. They intend to sell their remaining land if they are unable to meet the monthly debt repayment of US$12.5. She pays monthly US$16.5 for drugs and laboratory tests at a private provider."

**Loans with repayment in labour** are provided in cash but reimbursed in labour and termed *‘Yok Dai’* (taking hand). Respective borrowers are often very poor with limited ability to find enough cash to pay back. The loan is thus calculated in terms of the amount of daily work required, mostly farming work such as cutting or planting rice. The interest is hidden in the reduced value of labour which is repaid at below market prices. This type of loan is not everywhere practiced and related amounts tend to be small, often not more than a few dollars. Consequently, it is not so helpful for the poor to meet their health care costs, as these are often far higher.

"A very poor family consisting of a 15-year old orphan and his 68-year grandmother. The family has no regular income and relies on donations by relatives and neighbours. For treatment of the grandmother’s chronic illness they borrowed US$20 from a well-off farmer in the village. Since they had no income, the child had to work to repay the farmer. The daily payment for his labour was US$0.50 versus the prevailing market price of US$1 or more. Theoretically they could repay the US$20 within 40 days but, two years later, they had not been able to do since new loans were taken."

### Causes of health-related debt and coping mechanisms

The reported health problems leading to borrowing among the 47 households were a mixture of major infectious diseases (such as AIDS, dengue, typhoid fever, severe malaria, meningitis, severe injuries and acute abdominal syndromes) that require hospitalization, including major surgery, and chronic non-communicable diseases (such as diabetes, hypertension, heart diseases, asthma and cancer), which require lifelong treatment. Two respondents in Phnom Penh reported normal delivery as a cause of their indebtedness.

Care for infectious diseases and other acute problems was mainly sought from public hospitals. For chronic diseases, on the contrary, both public health facilities and private providers were consulted. Major expenses for chronic diseases were reported to stem from consulting private providers. The cost correlated with the number of providers consulted which resulted from shopping around for successful treatment. It is interesting to note that many of the indebted households were poor and eligible for HEF assistance; some did benefit from such assistance.

In response to the question of how would they pay off the debt, the large majority of the respondents said that they had no way to do so. For those who thought they were able to repay, most would resort to selling their last piece of land, working more or borrowing from relatives and friends.

## Discussion

The literature indicates that a large proportion of Cambodians borrow to meet the costs of health care. Access to formal credit suppliers, especially for the poor, is limited. Hence, they rely on loans with high interest rates from informal creditors. Consequently, many become heavily indebted with considerable potential for further impoverishment. A major reason for these borrowing practices is the limited cash flow in the Cambodian society. Prasso [28] pointed out that in 2000 the amount of money in circulation in the country stood at US$28 per person only. Historical reference is also made to the fact that French colonisers tried to tackle perverse borrowing practices by money lenders to peasants at annual interest rates of 200–300%. Current interest rates are lower though still enormous. In neighbouring Vietnam by contrast, monthly interest rates by private money lenders were found to be only 1.8% on average [29].

Many authors such as McIntyre *et al.*[2] and Storeng *et al.*[30] report on perverse effects of borrowing from money lenders to pay for health care related costs but do not mention interest rates associated with such loans. Since interest rates increase the amount of debt to be repaid, effects of such loans will last longer than for interest-free credit. Loans from money lenders are taken out because of the limited time frame during which health providers have to be paid. Borrowing from money lenders is also mostly practiced by people with limited social resources such as reciprocity between households or support from community organizations [2,31]. Social resources were reported to be one of the most important sources for quickly mobilising money although the poor tend to be the least likely to have access to them [32]. In Cambodia, the poorest have the least assets that can be quickly liquidated in tandem with limited social resources. As a result they will have to resort to money lenders and pay the highest interest rates because of lack of collateral.

As pointed out by Storeng and colleagues [30], a vicious cycle is created whereby loans are obtained to pay off interest of previous loans and assets are sold to pay off debt, which in turn further reduces productivity and thus income and ability to pay. But few studies have been done to assess the long term effects of loans for paying for health care on household livelihood [32]. The negative consequences of financial stress on mental health in low-income countries are increasingly being documented [33,34]. In India, for example, indebtedness is a major cause of suicide amongst farmers [35]. It is thus not unreasonable to advocate for longitudinal studies on coping strategies resulting from borrowing, especially with interest, to assess the long-term effects on household economies but also on health itself. Such studies could be done per type of informal credit and participants should be carefully selected since many resort to more than one credit source.

Strategies should be developed to limit borrowing with interest to pay for health care. One option could be to attract the population, especially the poor, to public health providers where costs are considerably lower than in the private sector [5]. A common strategy to attract patients to public health providers is to extend exemptions from user fees for certain population groups such as women and children, or for prevalent diseases of which control is a public good, such as tuberculosis [36]. Several authors have pointed out that free health care alone is not sufficient and that it also requires exemption initiatives to subsidise transport costs [37,38]. In Cambodia, HEF have been developed to attract the poor to exempted health services. They are extended nationwide as they greatly improve financial access [39]. However, the vast majority of beneficiaries of such schemes still first seek health care in the private sector where they incur most expenses for treating the condition [12,13]. Accordingly, at least in Cambodia, HEF unaccompanied by additional interventions are not the panacea to overcoming excessive borrowing costs from money lenders due to treatment seeking. Although HEF were not found to reduce the likelihood of incurring health-related debt, coverage by a HEF did reduce the amount of health-related debt by 25-28% [40]. More information is thus required on the determinants of care seeking to be able to attract people to the public sector when sick.

A more comprehensive approach than fee waivers or user fee exemptions is required, including interventions outside the health sector [31,41]. As such there was a call for more research on alternative health care financing strategies and mechanisms to enable improved coping with the costs of illness through reinforcement of existing social networks [2]. However, as seen above, the poor are least likely to access such networks. Nevertheless, with the emergence of chronic diseases and associated requirements for lifelong treatments in a financially constrained environment like Cambodia [42], the need for such community financing becomes more prominent [43]. Studies are therefore required to elicit existing barriers to social inclusion and means to overcome them.

Microfinance institutions have been advanced as a potential solution and they often go hand in hand with health interventions [44] to mitigate health problems and the need to spend money on related treatments. However, it has been reported from Bangladesh that the poorest women refused to participate in programs based on microcredit because of their aversion for the stress associated with loan repayments [45,46]. Others consider microfinance an effective risk mitigation strategy that enables borrowers to cope with sudden costs such as those associated with health care seeking [47]. The figures cited above indicate that the MFIs insufficiently penetrate rural Cambodia. Therefore, further research is needed to investigate whether extending microcredit to the poor can be used as a means to avert borrowing from informal creditors for health care expenses, and how this might be done.

If MFIs would be pursued as an option outside the health sector, additional interventions for the poorest should be implemented, similar to the experience in Bangladesh, including: provision of income generating asset grants, subsistence allowance until asset grants generate income, skills development training, social awareness development [41,45]. Alternatively, a potentially more powerful intervention for the poorest may be the provision of non-conditional cash transfers [30,34]. Meaningful research in Cambodia would then relate to identifying the most comprehensive approach to avoid or reduce the extent of borrowing from money lenders for health related expenses by the poor. This could consist of a combination of HEF and non-health sector interventions.

However, it is imperative that countries like Cambodia progress towards the development of regulatory interventions for the credit market. A package of basic consumer protection rights tailored to the problems faced by the poor in accessing money should be implemented [48].

## Conclusion

In Cambodia, similar to the situation in many other low-income countries where social health protection is underdeveloped, a large proportion of households have to borrow to meet the cost of health care. We paid special attention to the informal credit sources to which most cash-strapped patients or caretakers direct themselves to pay for treatment. Such credit practices create a vicious borrowing cycle for many households and induce further impoverishment. The findings suggest the importance of credit market failure and the insufficient social health protection system in Cambodia. Irrespectively of the type of *Changkar* used for obtaining money to pay for health care costs, economic (or livelihood) consequences appear considerable.

In order to successfully reduce borrowing practices from informal sources that charge exorbitant interest rates, a range of studies are suggested which would allow formulating the best strategy for this purpose. These assessments relate to longitudinal studies per type of *Changkar* and their effects on livelihoods and health of the borrowers and household members; eliciting care seeking determinants and gearing public sector practices towards the expectations of rural people in order to attract the poor to the public health providers, including identification of additional interventions to HEF to make them more effective. More information on strategies to overcome social exclusion, effective mechanisms for extension of MFIs to the poor and non-health interventions that can avert harmful borrowing practices is also required.

## Competing interests

We declare that we have no competing interests.

## Authors’ contributions

PI developed the study, collected and analysed the data, and wrote the research report on which this paper is based. In addition, he contributed to writing the final draft as well as revising and finalizing the accepted manuscript. BJ did the literature review and wrote the manuscript for submission and contributed to writing the revised and final version of this paper. BM and WVD provided content expertise throughout the design and implementation of the study, and contributed to the final version of the manuscript for submission. All authors read and approved the final manuscript.

## Pre-publication history

The pre-publication history for this paper can be accessed here:

http://www.biomedcentral.com/1472-6963/12/383/prepub

## References

[B1] Kruk ME Goldmann E Galea S Borrowing and selling to pay for health care in low- and middle-income countries Health Aff (Millwood ) 2009 28 1056 1066 10.1377/hlthaff.28.4.1056 19597204

[B2] McIntyre D Thiede M Dahlgren G Whitehead M What are the economic consequences for households of illness and of paying for health care in low- and middle-income country contexts? Soc Sci Med 2006 62 858 865 10.1016/j.socscimed.2005.07.001 16099574

[B3] Russell S Gilson L Are health services protecting the livelihoods of the urban poor in Sri Lanka? Findings from two low-income areas of Colombo Soc Sci Med 2006 63 1732 1744 10.1016/j.socscimed.2006.04.017 16766105

[B4] Leive A Xu K Coping with out-of-pocket health payments: empirical evidence from 15 African countries Bull World Health Organ 2008 86 849 856 10.2471/BLT.07.049403 19030690 PMC2649544

[B5] Cambodia Demographic and Health Survey 2010 2011 National Institute of Statistics, Ministry of Planning; Directorate General for Health, Ministry of Health; and ICF Macro, Phnom Penh, Cambodia

[B6] Murshid KAS Food Security in an Asian Transitional Economy: The Cambodian Experience 1998 Cambodia Development Resource Institute and United Nations Research Institute for Social Development, Phnom Penh

[B7] Domrei Research and Consulting Household debt in rural Cambodia 2011 Domrei Research and Consulting, Phnom Penh

[B8] Domrei Research and Consulting Health shocks and treatments in rural Cambodia 2011 Domrei Research and Consulting, Phnom Penh

[B9] Ir P Bigdeli M Meessen B Van Damme W Translating knowledge into policy and action to promote health equity: The Health Equity Fund policy process in Cambodia 2000–2008 Health Policy 2010 200 209 20189676 10.1016/j.healthpol.2010.02.003

[B10] Noirhomme M Meessen B Griffiths F Ir P Jacobs B Thor R Improving access to hospital care for the poor: comparative analysis of four health equity funds in Cambodia Health Policy Plan 2007 22 246 262 10.1093/heapol/czm015 17526640

[B11] Van Pelt M Morineau G Meessen B, Pei X, Criel B, Bloom G When slum dwellers seek health care: Exploring a community-based Health Equity Fund’s impact on indebtedness for health care and on utilisation of health services Health and social protection: experiences from Cambodia, China and Lao PDR 2008 Institute of Tropical Medicine, Antwerp, Belgium 491 518

[B12] Jacobs B Price NL Oeun S Do exemptions from user fees mean free access to health services? A case study from a rural Cambodian hospital Trop Med Int Health 2007 12 1391 1401 10.1111/j.1365-3156.2007.01926.x 17949399

[B13] Jacobs B Price N Meessen B, Pei X, Criel B, Bloom G A comparative study of the effectiveness of pre-identification and passive identification for hospital fee waivers at a rural Cambodian hospital Health and social protection: experiences from Cambodia, China and Lao PDR 2008 Institute of Tropical Medicine, Antwerp, Belgium 437 467

[B14] Besley T How do market failures justify interventions in rural credit markets? The World Bank Research Observer 1994 9 27 47 10.1093/wbro/9.1.27

[B15] Kenjiro Y Why Illness Causes More Serious Economic Damage than Crop Failure in Rural Cambodia Dev Chang 2005 36 759 783 10.1111/j.0012-155X.2005.00433.x

[B16] Gertler P Levine DI Moretti E Do microfinance programs help families insure consumption against illness? Health Econ 2009 18 257 273 10.1002/hec.1372 18634128

[B17] Daru P Churchill C Beemsterboer E The prevention of debt bondage with microfinance-led services Eur J Development Research 2005 17 132 154 10.1080/09578810500066704

[B18] Van Damme W Van Leemput L Por I Hardeman W Meessen B Out-of-pocket health expenditure and debt in poor households: evidence from Cambodia Trop Med Int Health 2004 9 273 280 10.1046/j.1365-3156.2003.01194.x 15040566

[B19] Oxfam Phnom Penh 2000 Oxfam, Phnom Penh, Cambodia

[B20] World Bank GDP per capita (in current USD) http://data.worldbank.org/indicator/ NY.GDP PCAP.CD, accessed 22.01.2012.

[B21] World Bank Poverty profile and trends in Cambodia in 2007. Findings from the Cambodia Socio-Economic Survey (CSES) 2009 World Bank, East Asia and the Pacific, Bangkok

[B22] Ballard BM Sloth C Wharton D We are living with worry all the time 2007 Cambodia Development Resource Institute, A participatory poverty assessment of the Tonle Sap. Phnom Penh

[B23] Meessen B Bigdeli M Chheng K Decoster K Ir P Men C Composition of pluralistic health systems: how much can we learn from household surveys? An exploration in Cambodia Health Policy Plan 2011 26 Suppl 1 i30 i44 21729915 10.1093/heapol/czr026

[B24] Kem S Policy Options for Vulnerable Groups: Income Growth and Social Protection 2011 Cambodia Development Resource Institute, Council for Agricultural and Rural Development and International Food Policy Research Institute, Phnom Penh

[B25] Phlong P Informal credit systems in Cambodia 2009 Department of Anthropology, Northern Illinois University, Illinois

[B26] Meessen B Zhenzhong Z Van Damme W Devadasan N Criel B Bloom G Iatrogenic poverty Trop Med Int Health 2003 8 581 584 10.1046/j.1365-3156.2003.01081.x 12828538

[B27] Watts JJ Segal L Market failure, policy failure and other distortions in chronic disease markets BMC Health Serv Res 2009 9 102 10.1186/1472-6963-9-102 19534822 PMC2704185

[B28] Prasso DST The Riel value of money: how the world’s only attempt to abolish money has hindered Cambodia’s economic development. Asia Pacific Issues 49 2011 East–west Center, Honolulu

[B29] Barslund M Tarp F Formal and informal credit in four provinces of Vietnam J Development Studies 2008 44 485 503 10.1080/00220380801980798

[B30] Storeng KT Baggaley RF Ganaba R Ouattara F Akoum MS Filippi V Paying the price: the cost and consequences of emergency obstetric care in Burkina Faso Soc Sci Med 2008 66 545 557 10.1016/j.socscimed.2007.10.001 18061325

[B31] Molyneux C Hutchison B Chuma J Gilson L The role of community-based organizations in household ability to pay for health care in Kilifi District, Kenya Health Policy Plan 2007 22 381 392 10.1093/heapol/czm031 18006525

[B32] Russell S The economic burden of illness for households in developing countries: a review of studies focusing on malaria, tuberculosis, and human immunodeficiency virus/acquired immunodeficiency syndrome Am J Trop Med Hyg 2004 71 147 155 15331831

[B33] Lund C Breen A Flisher AJ Kakuma R Corrigall J Joska JA Poverty and common mental disorders in low and middle income countries: A systematic review Soc Sci Med 2010 71 517 528 10.1016/j.socscimed.2010.04.027 20621748 PMC4991761

[B34] Lund C De SM Plagerson S Cooper S Chisholm D Das J Poverty and mental disorders: breaking the cycle in low-income and middle-income countries Lancet 2011 378 1502 1514 10.1016/S0140-6736(11)60754-X 22008425

[B35] Behere PB Behere AP Farmers’ suicide in Vidarbha region of Maharashtra state: A myth or reality? Indian J Psychiatry 2008 50 124 127 10.4103/0019-5545.42401 19742218 PMC2738339

[B36] Witter S Service- and population-based exemptions: are these the way forward for equity and efficiency in health financing in low-income countries? Adv Health Econ Health Serv Res 2009 21 251 288 19791706

[B37] Kruk ME Mbaruku G Rockers PC Galea S User fee exemptions are not enough: out-of-pocket payments for ‘free’ delivery services in rural Tanzania Trop Med Int Health 2008 13 1442 1451 10.1111/j.1365-3156.2008.02173.x 18983268

[B38] Witter S Removal of user fees for health care - a review of recent experiences in Africa 2010 The World Bank, Khartoum

[B39] Tangcharoensathien V Patcharanarumol W Ir P Aljunid SM Mukti AG Akkhavong K Health-financing reforms in southeast Asia: challenges in achieving universal coverage Lancet 2011 377 863 873 10.1016/S0140-6736(10)61890-9 21269682

[B40] Flores G Ir P Men CR O’Donnell O van Doorslaer E Financial protection of patients through compensation of providers: the impact of health equity funds in Cambodia 2011 Tinbergen Institute Discussion Paper, Amsterdam 10.1016/j.jhealeco.2013.09.012 24189447

[B41] Ahmed SM Petzold M Kabir ZN Tomson G Targeted intervention for the ultra poor in rural Bangladesh: Does it make any difference in their health-seeking behaviour? Soc Sci Med 2006 63 2899 2911 10.1016/j.socscimed.2006.07.024 16954049

[B42] Ir P Men C Lucas H Meessen B Decoster K Bloom G Self-reported serious illnesses in rural Cambodia: a cross-sectional survey PLoS One 2010 5 e10930 10.1371/journal.pone.0010930 20532180 PMC2880606

[B43] Beaglehole R Epping-Jordan J Patel V Chopra M Ebrahim S Kidd M Improving the prevention and management of chronic disease in low-income and middle-income countries: a priority for primary health care Lancet 2008 372 940 949 10.1016/S0140-6736(08)61404-X 18790317

[B44] Leatherman S Metcalfe M Geissler K Dunford C Integrating microfinance and health strategies: examining the evidence to inform policy and practice Health Policy Plan 2012 27 2 85 101 10.1093/heapol/czr014 21343235

[B45] Halder SR Mosley P Working with the ultra-poor: learning from BRAC experience J Int Dev 2004 16 387 406 10.1002/jid.1084

[B46] McIntyre L Rondeau K Kirkpatrick S Hatfield J Islam KS Huda SN Food provisioning experiences of ultra poor female heads of household living in Bangladesh Soc Sci Med 2011 72 969 976 10.1016/j.socscimed.2011.01.011 21345564

[B47] Mohindra K Haddad S Narayana D Can microcredit help improve the health of poor women? Some findings from a cross-sectional study in Kerala, India Int J Equity Health 2008 7 2 10.1186/1475-9276-7-2 18186918 PMC2254417

[B48] Brix L McKee L Consumer protection regulation in low-access environments: opportunities to promote responsible finance 2010 CGAP, Washington

